# Supplementary material for: Improving Environmental Capacities for Health Promotion in Support Settings for People with Intellectual Disabilities: Inclusive Design of the DIHASID Tool
Source: Int J Environ Res Public Health. 2020 Jan 28;17(3):794. doi: 10.3390/ijerph17030794 (PMC7037294; doi:10.3390/ijerph17030794)
Supplement: Supplementary file 1 [file ijerph-17-00794-s001.pdf]

The survey can be used by clients, client representatives, care professionals, and team leaders at ID support organizations.
For convenience and clarity, the questions in the online survey are automatically adapted to the target audience. There are six types of surveys available in Dutch. Three for residential support centers and three for daytime support centers. For each location, there is one for clients, one for client representatives, and one for care professionals and team leaders.

This version of the survey is a combination of them, and the differences between the six surveys are as follows:
→ Per target group and location, the different options are provided in square brackets [...], for the client, client representative, and care professionals or team leader respectively.
Example: How old [are you/is the person you represent/are the clients]?
→ Some questions do not need to be answered by clients and/or client representatives. Those questions are marked with “*... only*”.
*Example: V1. *Representatives only* What is your relationship with the resident or participant in the daytime care activities on whose behalf you are filling out this form?*

Healthy environment survey

The images in this survey are used under the Creative Commons Licence 2.0. The colored pictures are made by the author. The black and white pictures are from Sclera.be and the following contributors to the Noun project; Andrew Doane, Hea Poh Lin, Alvaro Cabrera, Corpus Delicti, John T. Garcia, Yu Luck, Parkjisun, Gan Khoon Lay, Björn Andersson, Luis Prado, Gan Khoon Lay, Studio Het Mes.

This survey is about healthy living.

Healthy living is:

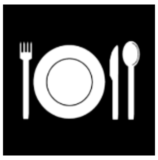

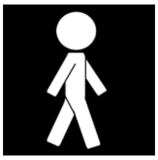

Healthy food and drink   Physical activity and sports

Complete the questions for a residential or a daytime care location.

The survey consists of 4 parts:

1. Questions about you (or the person/persons you represent).
2. How people help with healthy living.
3. How places help with healthy living.
4. How plans and money from the organization help with healthy living.

Everyone must complete parts 1, 2, and 3. Part 4 only needs to be completed by representatives and care professionals.

There are 3 types of questions in the survey.
Questions about what already exists, what you think about it, and what your wishes and dreams are.
When you write about what already exists, you may think of things that you want. Write those things down for the questions about dreams and wishes.

If you do not know the answer to a question, check "I do not know".
If the question does not apply to you/your client(s), check "not applicable".
For example, the question about talking with a client if they are unable to speak.

Part 1: General questions

1. I am:

Check one box.

- o A client.
- o A person filling in the list on behalf of one client.
- o An attendant.

Care professionals only When you complete the survey, think about all clients you care for in this location.

B1 *Care professionals only* **How many clients are there at the residential or daytime care group?**

V1 *Representatives only* **What is your relationship with the resident or participant in the daytime care activities on whose behalf you are filling out this list?**

- o Parent
- o Brother/sister
- o Daily attendant
- o Other:

2. [I am a/The person on whose behalf I am filling out the survey/The client group consists of]:

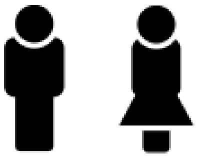

○ Man ○ Woman

3. How old [are you/is the person you represent/are the clients]?

4. I am completing this survey for:

Check one of the boxes

- A home. The building is in a residential area.
- A home. The building is on the grounds of a care organization complex.
- A daytime care location or workplace. The building is in a residential area.
- A daytime care location or workplace. The building is on the grounds of a care organization complex.

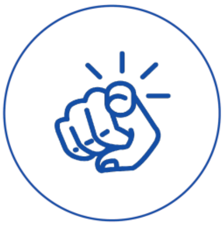

5. [Do you have/Does the person you represent have/Do one or more clients have] any of the following disabilities?

Check all that apply.

- 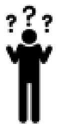 I do not know. Or: I prefer not to say.
- 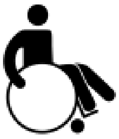 [I use/The person I represent uses/One or more clients use] a wheelchair. [I can move myself/The person I represent can move themselves/One or more clients can move themselves] in a manually operated wheelchair.
- 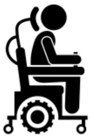 [I use/The person I represent uses/One or more clients use] a wheelchair. [I can move myself/The person I represent can move themselves/One or more clients can move themselves] in an electric wheelchair.
- 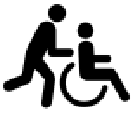 [I use/The person I represent uses/One or more clients use] a wheelchair, and someone else needs to push the chair.
- 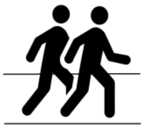 [I am/The person I represent is/One or more clients are] not allowed on the road by [myself/themselves/themselves].
- 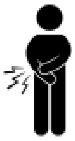 [I have/The person I represent has/One or more clients have] spasms.
  Example: you have an arm or a leg that you cannot fully use. Because of stiff muscles or muscles that suddenly contract.
- 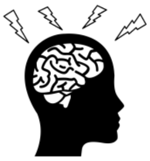 [I have/The person I represent has/One or more clients have] epilepsy. *Example: your brain can short-circuit. When this happens you will notice things like strange movements, convulsions, falling down, or even falling unconscious.*

○ 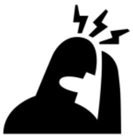 [I have/The person I represent has/One or more clients have] difficulty processing stimuli (autism).
Example: You cannot deal with a lot of noise or lots of people around you.

○ 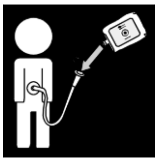 [I have/The person I represent has/One or more clients have] a feeding tube.
Example: You receive food through a tube in your stomach.

○ 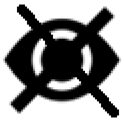 [I have impaired vision or am blind/The person I represent has impaired vision or is blind/One or more clients have impaired vision or are blind.]
Even when wearing glasses.

○ 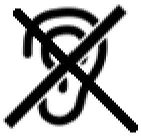 [I am hard of hearing/The person I represent is hard of hearing/One or more clients are hard of hearing.]
Even when wearing a hearing aid.

○ 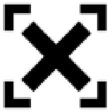 [I have/The person I represent has/One or more clients have] no impairments.

○ Other:

Part 2: People

Healthy living is:

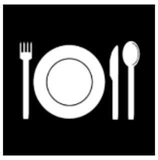

Healthy food and drink

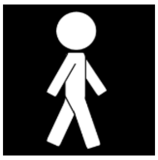

Physical activity and sports

Maybe different people support you.
This question is only about the people who help you with healthy living.

6. Who helps [you/the person you represent/the clients] [in daytime care/at home] with healthy living?

Check all that apply.

- Care professionals
- Family
- Clients or fellow residents
- Friends
- Volunteers
- A care professional who specifically helps with healthy living.
  Explanation: Healthy living awareness specialist
- Doctor
- Physiotherapist
  Explanation: This is someone who helps with specific exercises for movement.
- Exercise specialist
  Explanation: Someone who helps with physical activities and exercise.
- Occupational therapist
  Explanation: Someone who gives you exercises or helps with adapting your home. Things such as adding a chair in the shower or adjusting the height of the kitchen.
- Masseur
  Explanation: Someone who relaxes your muscles.
- Dietician
  Explanation: Someone who gives you tips about healthy food and drink.
- Speech therapist
  Explanation: Someone who can help when eating and drinking is difficult.
- Someone else, answer: ____________

- I do not know. Or I prefer not to say.

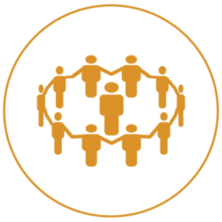

BV1 *Representatives and care professionals only* **Do care professionals at this location have time to motivate the clients to be physically active?**

- Never
- Sometimes
- Often
- Always
- I do not know

BV2 *Representatives and care professionals only* **Do care professionals at this location have time to sufficiently focus on food and provide the clients with a peaceful time to eat?**

- Never
- Sometimes
- Often
- Always
- I do not know

7. **What do the people [in the daytime care/in the home] do together?**

This question is about what [you do/the person you represent does/the clients do] together with the clients and/or care professionals.
You do not need to check off the things [you do/the person you represent does/the clients do] alone.

Check all that apply.

- Sports.
- Physical activity.
  Example: walking, cycling, mat exercises, and exercising to music.
- Staying active throughout the day.
  Example: getting a cup of coffee, taking out the trash, cleaning, dressing oneself, controlled MSE, and playing.
- Grocery shopping.
- Cooking.
- Eating together.
- Talking about healthy living.
- Making agreements about healthy living.
  Example: You agree with your care professional that you will eat one piece of fruit every day.
- Nothing that has to do with healthy living.
- I do not know.
- Other: ______

Healthy living is:

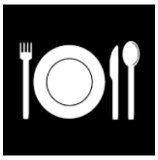

Healthy food and drink

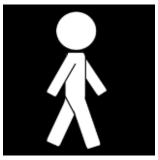

Physical activity and sports

8. How do others help [you/the person you represent/the clients] [at daytime care/in the home] with healthy living?

Check all that apply.

- o Encourage healthy living.
  Example: your care professional says "have an apple today!"
- o Explain things about healthy living.
- o Support by giving tips about healthy living.
- o Show others what healthy living looks like.
  Example: your care professional eats a healthy lunch.
- o Help with going to an exercise activity.
- o Help with getting dressed for sports.
- o Help choosing an exercise or sports activity.
- o Help choosing food and drink.
  Example: creating a menu together, or offering clients two types of vegetables to choose from.
- o Others buy healthy food and drink.
  Example: grocery shopping or ordering food.
- o Serve food and pour drinks.
- o Others prepare breakfast, lunch, and/or dinner.
- o The other people here do not help with healthy living.
- o I do not know.
- o Other: ______

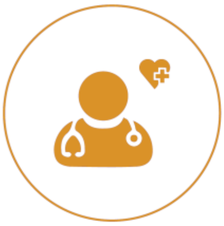

People who know a lot about healthy living can also help.
It could be the doctor, physiotherapist, exercise specialist, occupational therapist, dietician, speech therapist, etc.

9. How do these people help [you/the person you represent/the clients] with healthy eating, healthy drinking, exercise, and sport?
Check all that apply.

- Exercise activities.
  Example: fitness and swimming
- Movement exercises.
  Example: from the physiotherapist
- Assisted exercise.
  Example: when you cannot move your leg, and someone else moves it for you.
- Information and tips about exercising.
  Example: The physiotherapist tells you what sports you can do.
- Information and tips about healthy food and drink.
  Example: The dietician tells you which food is healthy.
- Cooking classes.
- Giving advice when healthy eating is difficult.
  Example: If swallowing is difficult, eating too much or not enough.
- I do not receive this help.
- I do not know.
- Other: ______

B2a *Care professionals only* **At this location, there is enough opportunity for care professionals to get tips or advice about...:**

- Exercise aids.
- Exercise options and motivating clients.
- Ways you can offer support for the exercises the clients have received from a physiotherapist or exercise specialist.
- Inspiring materials you can use (exercise folder, exercise bag) to motivate clients to engage in physical activity.
- Inspiring materials you can use (such as cooking workshops, videos, menus) to create healthy meals.
- Ways to make eating easier for clients with problems swallowing.
- Ways food can be fine-tuned to what the clients need.
- There are no opportunities for this.
- I do not know.

B2b *Care professionals only* **Who is available to provide this advice?**

- Physiotherapist
- Exercise specialist
- Occupational therapist
- Speech therapist
- Dietician
- General practitioner (GP)
- Intellectual disability physician
- I do not know
- Other:

10–12. To what extent do the following people help with healthy living?

Check the boxes that are most applicable.

	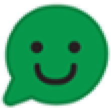 Good	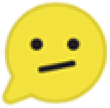 Could be better	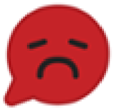 Bad	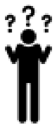 I do not know
Care professionals, clients, and volunteers				
Family and friends				
People who know a lot about healthy living*				

**People who know a lot about healthy living include: doctors, physiotherapists, exercise specialists, occupational therapists, dieticians, and speech therapists.*

Care professionals only B3. **Do care professionals receive assistance from healthcare professionals in the area of healthy living?**

- o Very unsatisfactory
- o Unsatisfactory
- o Moderate
- o Satisfactory
- o Good
- o I do not know

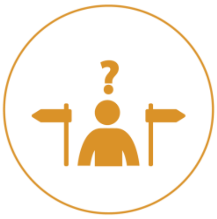

Healthy living is:

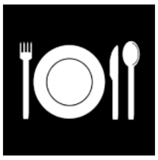 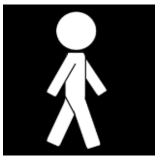

Healthy food and drink   Physical activity and sports

13. How do others help [you/the person you represent/the clients] make choices about healthy living?

Check what applies to you. You can check more than one box.

- o Clients choose themselves; they do not receive help.
- o Clients choose themselves; others give tips.
  Example: care professional gives tips about what healthy drinking is; client chooses what they will do.
- o Care professionals and clients choose together.
  Example: creating a weekly menu together or choosing a sport.
- o Care professionals say what you can choose from. Clients choose what they want.
  Example: Care professional shows two vegetables. Client indicates what they want.
- o Care professionals consider client preferences.
  Example: Care professional chooses to take walks with clients. The care professional knows that the clients will like this.
- o Care professionals make the choice for the client.
  Example: Care professional makes the choice, as the client is not allowed to do so or capable of doing so.
- o I do not know.

14. What do you think about the help [you receive/the person you represent receives/the client receives] in relation to <u>making personal choices.</u>

For example, choices about exercise, sports, healthy food, and drink?

Check the box below the smiley face that is the best match.

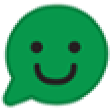	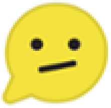	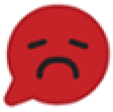	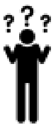	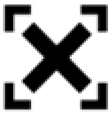
o	o	o	o	o
Good	Can be	Bad	I do not know	Not applicable

improved

Healthy living is:

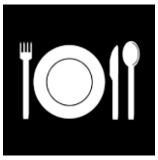

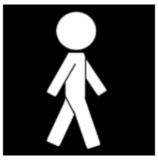

Healthy food and drink   Physical activity and sports

15. [At day care/in the residential group], clients and care professionals talk with each other about healthy living.

Check the best match:

- o Never
- o Sometimes
- o Often
- o Always
- o I do not know
- o Not applicable

16. What do you think about the way they talk about healthy living at the [daytime care group/residential group]?

Check the box below the smiley face that is the best match.

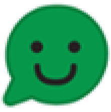

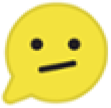

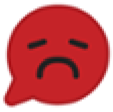

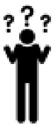

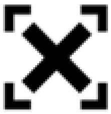

o	o	o	o	o
Good	Can be improved	Bad	I do not know	Not applicable

When we say healthy living, we mean healthy food, healthy drink, sufficient exercise and sports.

BV3–7 To what extent do you agree with the following statements about care professionals at this location:

	Completely disagree	Disagree	Neutral	Agree	Completely agree	I do not know
The team of care professionals has sufficient knowledge and skills in relation to healthy living.	○	○	○	○	○	○
The team of care professionals has sufficient knowledge about every client so that personalized support can be offered for healthy living. For example, the team knows what type of exercise is beneficial for a client with spasms.	○	○	○	○	○	○
The team has clear mutual agreements about supporting clients in relation to healthy living.	○	○	○	○	○	○
The team has clear agreements with the family of clients about providing support in relation to healthy living.	○	○	○	○	○	○
The team has a shared vision of healthy living.	○	○	○	○	○	○

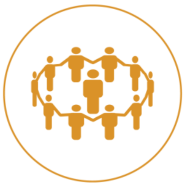

People around you

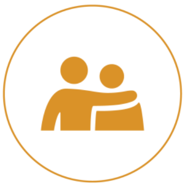

Help from others

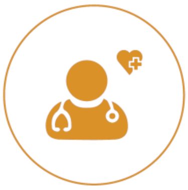

Healthcare

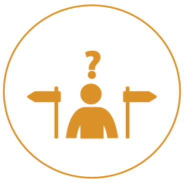

Supporting own choices

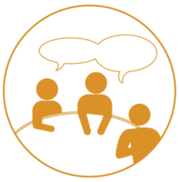

Open discussion

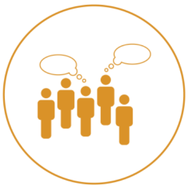

Thinking about healthy

living

A17. You have told us about how people [at daytime care/in the home] help with healthy eating, drinking, exercise, and sports.

<u>How can people help even more with healthy living?</u>

<u>What are your dreams, tips, or ideas?</u>

Clients only *You can write something in the box below. Maybe someone can help you.*

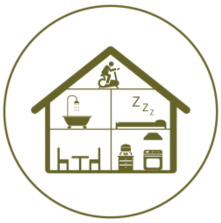

PART 3: Places and Aids

18. Which things (aids) for exercise are available [at daytime care/in the home]?

Check all that apply.

- Yard in which you can do exercises.
- Enough space inside to do exercises.
- A hall or space for engaging in physical activity.
  Example: gymnasium or fitness space at the daytime care location.
- Multisensory environment (MSE room).
- Stationary bicycle.
- Bicycle or buddy bicycle.
- Bicycle for the wheelchair.
  Example: a MOTOmed, Thera Trainer, or Theravital.
- Book with ideas about exercise activities.
- Exercise equipment.
  Example: exercise bag, mat, and jump rope.
- Games in which you need to move.
  Example: Wii or interactive games.
- Patient lift. This is a special device for lifting someone from a wheelchair.
- Building without thresholds.
- Wide doors.
  Example: The doors are wide enough for someone in a wheelchair.
- *We do not have the things mentioned above.*
- I do not know.
- Other:

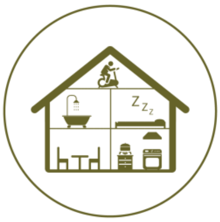

19. Which things (aids) for healthy eating and drinking are available [at daytime care/in the home]?

Check all that apply.

- o Kitchen and cooking supplies.
- o Meal service.
  Explanation: ready-made meals are delivered.
- o Meal-in-a-box or groceries are provided.
  Explanation: items are supplied that can be used to make food.
- o Vegetable garden or fruit trees.
- o Healthy food and drink at home.
- o Foods list
  Explanation: a list of the food and drink that everyone likes.
- o Recipe book
- o We do not have the things mentioned above.
- o I do not know.
- o Something else.
  Answer:

Healthy living is:

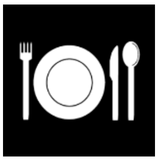

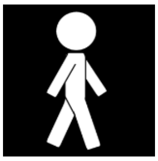

Healthy food and drink   Physical activity and sports

20. How do the things (aids) fit in with what [you need/the person you represent needs/the clients need] for healthy living?

Check the box below the smiley face that is the best match.

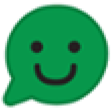

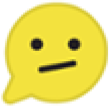

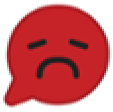

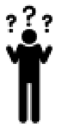

o Good	o Can be improved	o Bad	o I do not know

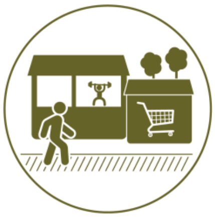

With “near”, we mean on the site and/or in the area around [the home/the daytime care location].

21. Which of these are near [the daytime care/the home]?

Check all that apply.

- [Home/Daytime support center]
- Supermarket
- Shops
- Swimming pool
- Hydrotherapy bath
  Explanation: this is a water therapy bath.
- Riding stables
  Explanation: you can ride horses here.
- Gymnasium, sports hall, or fitness space
- Playground or outdoor exercise area
- Sports field
- Grounds for walking and cycling
- Green space for walking or cycling
  Example: walking in a park or forest
- There are no places nearby that can help people with healthy living.
- I do not know

22. How far is...

For each location, check the box that is best for you/your client(s).

	You can walk there (within 15 minutes)	You can cycle there (within 15 minutes)	You need a car, taxi, or bus to get there.	I do not know
[Home/Daytime support center]	○	○	○	○
Supermarket	○	○	○	○
Shops	○	○	○	○
Swimming pool	○	○	○	○
Hydrotherapy bath	○	○	○	○
Riding stables	○	○	○	○
Gymnasium, sports hall, or fitness space	○	○	○	○
Playground or outdoor exercise area	○	○	○	○
Sports field	○	○	○	○
Area for walking and cycling	○	○	○	○
Green space for walking or cycling	○	○	○	○

By “near”, we mean on the site of and/or in the area around [the home/the daytime support center].

23. **What do you think of the locations near [the daytime care location/your home] with regard to:**

	Good	Could be better	Bad	I do not know
... healthy food and drink				
... physical activity and sport				

25. What do you think about the <u>activities</u> for healthy living that [you/the person you represent/clients] can participate in?

Example: You can participate in cooking lessons and fitness.

Check the box below the smiley face that is the best match.

o Good	o Can be improved	o Bad	o I do not know

BV8 *Attendants + representatives only* **Is healthy living sufficiently woven into the day and evening program?**

Check the box below the smiley face that is the best match.

o o o o

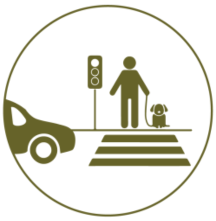

Good	Can be improved	Bad	I do not know

By “near”, we mean on the site of and/or in the area around [the home/the daytime care location].

26. How safe [do you feel/does the person you represent feel/do your clients feel] to go to nearby places?

Check the box below the smiley face that is the best match.

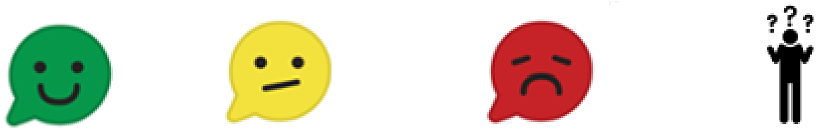

o	o	o	o
Safe	Can be safer	Needs to be safer	I do not know

27. How easy is it to go to nearby places?

Check the box below the smiley face that is the best match.

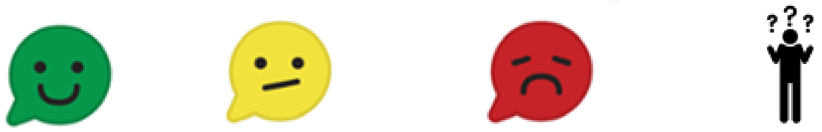

o	o	o	o
Easy	Could be easier	Needs to be easier	I do not know

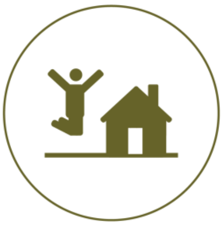

Healthy living is:

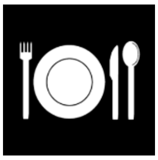

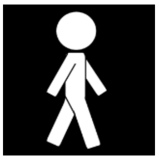

Healthy food and drink   Physical activity and sports

28. Think about [what you need/what the person you represent needs/the clients need] for healthy living. Is that available <u>at</u> the [home/daytime care location]?

Check the box below the smiley face that is the best match.

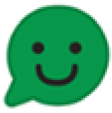
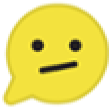
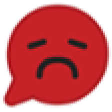
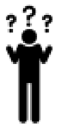

o	o	o	o
Enough	Could be more	Needs to be more	I do not know

29. Think about [what you need/what the person you represent needs/the clients need] for healthy living. Is that available <u>near</u> the [home/daytime care location]?

Check the box below the smiley face that is the best match.

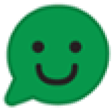	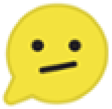	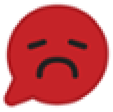	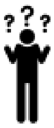
o	o	o	o
Enough	Could be more	Needs to be more	I do not know

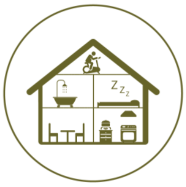

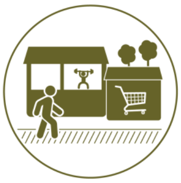

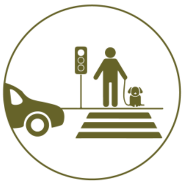

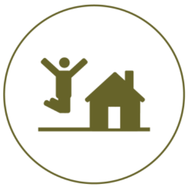

Healthy home environment *Locations for healthy activities* *Ease of travel* *Tailored*

You have told us about how places help with healthy food, drink, physical activity, and sport.

30. What is still needed at the [daytime care location/home] **or nearby areas for healthy living?**
What are your dreams, tips, or ideas?
*****the following instructions for clients only* You can write something in the box below.*

Maybe your care professional can help you.

Clients only **C1. Are there other things that can be improved?**

Care professionals are also asked questions about money and the organization's plans.
Do you also want to add something about how money and the organization's plans can help with healthy living?

Write it down below.

Part 4: Policy and budget for healthy living

BV9–12 *Representatives and care professionals only* **The following questions are about financial resources.**

	Very unsatisfactory	Unsatisfactory	Moderate	Satisfactory	Good	I do not know
Do the clients at this location have enough money to spend freely on exercise activities, exercise materials, movement aids, and healthy food?	○	○	○	○	○	○
Does this location have enough money to spend on healthy food and tools for healthy foods (such as kitchen utensils)?	○	○	○	○	○	○
Does this location have enough money to spend on physical activity, exercise equipment, and movement aids for clients?	○	○	○	○	○	○
Do you feel that the care organization has reserved enough in the budget and in personnel capacity to support clients with healthy living?	○	○	○	○	○	○

BV13 *Representatives and care professionals only* **What do you think about the <u>financial resources</u> for healthy living at this location?**

- Very unsatisfactory
- Unsatisfactory
- Moderate
- Satisfactory
- Good
- I do not know

BV14–22 *Representatives and care professionals only* **The following questions are about the organization's policy.**

	Very unsatisfactory	Unsatisfactory	Moderate	Satisfactory	Good	I do not know
What do you think about the attention paid to healthy living in the organization's policy?	○	○	○	○	○	○
What do you think about the attention paid to healthy living in the organization's communications?	○	○	○	○	○	○
To what extent does the organization align and collaborate with municipalities in relation to spatial planning and sports providers (for example, a sports service desk)?	○	○	○	○	○	○
To what extent does the policy take the wishes of the different target groups into consideration?	○	○	○	○	○	○
To what extent are clients actively involved in the creation of a healthy living environment at the locations?	○	○	○	○	○	○
To what extent is there attention for exercise and healthy food in the discussions of the client's development plan*?	○	○	○	○	○	○
Does the organization determine what knowledge employees and clients need to have in relation to lifestyle?	○	○	○	○	○	○
To what extent are care professionals provided with knowledge and coaching from other employees within the organization to support clients with healthy living?	○	○	○	○	○	○
To what extent can care	○	○	○	○	○	○

professionals make use of coaching by external parties, courses, and education in relation to providing support for healthy living?						

** Some organizations call the development plan a care plan, individual guidance plan, support plan, or treatment plan*.

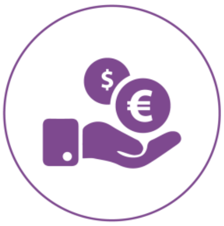

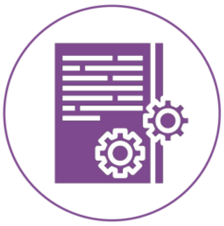

BV23 *Representatives and care professionals only* You have just told us about the situation at this location in relation to policy and budget for healthy eating, healthy drinking, sufficient exercise, and sport.
Do you have tips or ideas? What is needed in the policy? Which financial resources are needed?
